# ‘I think from my experience, it’s a negative thing for most people’: Exploring the perspectives of young British women opposed to the calories on menus policy in the UK

**DOI:** 10.1177/13591053251318192

**Published:** 2025-02-19

**Authors:** Maya Canzini, Jasmin Langdon-Daly

**Affiliations:** University of Bath, UK

**Keywords:** calories on menus, disordered eating, obesity prevention policy

## Abstract

The compulsory inclusion of calorie information on menus has been health policy in the UK since 2022. Public opinion on the policy varies, with young women particularly likely to oppose and express concerns. This qualitative study explores the perspectives of young women with a negative opinion of the policy. Eight White British women (18–25 years) volunteered to take part in semi-structured interviews exploring their perceptions and experiences with calories on menus. Inductive reflexive thematic analysis developed three themes: (1) viewing calories fed their own unhealthy relationship with food; (2) calories don’t tell you everything that matters about food; (3) negative emotional reactions are shaped by society’s encouragement of the thin ideal. The women’s negative opinions on the policy appeared heavily grounded in, and indicative of, their own negative emotional responses to viewing calorie information. They linked these responses to sociocultural meanings and ideals related to thinness, food choice and dieting.

## Introduction

High rates of obesity are considered a significant public health issue, both nationally in the UK and on a global scale, resulting in significant impacts on healthcare systems, government finances, general productivity and the mental wellbeing of the population (Dobbs and Manyika, 2015). Levels of obesity continue to rise with statistics showing that since 1933, the proportion of individuals in the UK who are overweight or obese has risen from 52.9% to 64.3% and those who are obese from 14.9% to 28.0% (Baker, 2023). The causes of this drastic increase are multifaceted and not fully understood (Hill and Melanson, 1999). The aetiology involves interactions between genetic, environmental, physiological, psychological, social and economic factors (Aronne et al., 2009). Some factors likely to have played a role include foods with a higher caloric value being more affordable and easily accessible (Rolls, 2003), a decrease in physical activity (Garber, 2019; Stockwell et al., 2021), and an increase in sedentary behaviours (Andersen et al., 1998), alongside other factors (Keith et al., 2006).

Alongside rising rates of obesity, in recent years there has been a reported increase in rates of mental health problems in the UK population, especially amongst young adults (Lee et al., 2020). This includes more individuals suffering from depression (Hawes et al., 2021), anxiety (Fardin, 2020) and eating disorders (Taquet et al., 2021). Eating disorders can be defined as serious psychological and physical disorders characterised by persistent disturbance of eating behaviours and accompanying distress, causing significant impairment to health and psychosocial functioning (American Psychiatric Association, 2013). Disorders such as anorexia nervosa and bulimia nervosa are most common amongst adolescent girls and young women aged 16–25 (Garner et al., 1980). Disordered eating behaviours (unhealthy weight control behaviours and significant poor body image) are very common amongst girls and women in this age group (Croll et al., 2002). Socio-cultural westernised ideals and discourses related to valuing slimness and ‘healthy’ or ‘clean’ eating, and denigrating and fearing ‘fatness’ and overweight, have been implicated in the development of these difficulties (Rodgers, 2016; Rodgers et al., 2022). Young women appear to be particularly subject to these discourses (Woolhouse et al., 2012).

The UK government announced in 2020 that they would be implementing a new anti-obesity strategy as it was believed there was a significant relationship between weight and the increased risk and severity of COVID-19 (Kwok et al., 2020). This strategy included larger businesses serving food to include the calorie information on menus (Department of Health and Social Care, 2021) as an incentive to reduce obesity. The impact of this policy on eating behaviour is still being investigated, but studies suggest that the introduction of this policy has not led to a significant reduction in calories ordered (Polden et al., 2024).

Whilst this aims to benefit the public, concerns were raised about a lack of consideration of potential negative impacts to consumers, including groups at higher risk of disordered eating and eating disorders, such as young women (Beat, 2020). Calorie counting is present in many eating disorders and is strongly associated with eating concerns and dietary restraints (Simpson and Mazzeo, 2017). Larson et al. (2018) found significant associations between women’s reported use of menu labels to reduce calorie intake and an increased number of self-reported weight concerns. Increased use of calorie labels was also associated with increased unhealthy weight-control behaviours including fasting, self-induced vomiting, and the use of laxatives and diuretics, as well as greater binge-eating. While causality cannot be inferred, it is plausible that viewing calorie information readily available on menus could activate or amplify meanings and anxieties related to unhelpful ‘clean eating’ or ‘fear of fatness’ norms and scripts, potentially increasing anxiety, disordered eating or poor body image experiences in individuals most subject to these norms (e.g. young women).

Whilst there is a variety of research linking calorie counting to eating disorders, body dissatisfaction, and generally negative implications on mental health (Haynos and Roberto, 2017; Embacher Martin et al., 2018; McGeown, 2019), fewer studies have investigated the impact of the introduction of calories on menus and how this has affected the public. Lillico et al. (2015) found no increase in questionnaire measures of disordered eating following the introduction of calorie information to the menu in a university canteen. One experimental study found that viewing calorie information and disordered eating status did not impact on food choice or calories consumed (Shoychet et al., 2023). In contrast, a similar experimental study identified an interaction of eating disorder status and calorie information, with individuals reporting symptoms consistent with anorexia or bulimia nervosa ordering less calories after viewing this information, while those reporting symptoms of binge eating disorder ordered more (Haynos and Roberto, 2017).

Whilst all groups may be impacted, whether positively or negatively, by the introduction of calories on menus, specific groups may feel this impact differently. Recent research has investigated the impact of calorie menu information on specific ‘at risk’ groups. In a survey of sexual minority men in the USA, participants who reported disordered behaviours were more likely to notice calorie information on menus than those who did not, and those that did notice calorie information were more likely to order less calories as a result of the information (Salvia et al., 2024). A large-scale qualitative survey of individuals with a diagnosis of anorexia in the UK described their views that calories on menus were detrimental to their eating disorder, increased their social isolation, restricted their freedom and left them feeling upset and angry. These social impacts make sense given the sociological importance of eating out as an enjoyable and pleasurable social experience (Warde and Martens, 2000).

In the context of limited research into this area, a significant public discourse remains active about the negative impact of calories on menus, particularly around increasing the prevalence of eating disorders or impacting on those who have eating disorders (Polden et al., 2023; Putra et al, 2023; Robson, 2024). Young women in particular appear more likely to endorse a negative view of the policy and raise concerns related to eating disorders (YouGov, 2022). In a survey, half of young women (51%) consulted opposed the policy, and 72% thought it likely to be harmful to those with eating disorders, compared to 33% and 37% respectively for the UK public overall. These negative views are interesting in the context of young women (aged 16–25) being at highest risk for the onset of eating disorders, and potentially more subject to sociocultural ideals and meanings related to body image, weight, and ‘healthy eating’. The nature of young British women’s concerns about calories on menus have not yet been explored and described in research.

### Aims and objectives

This study aims to explore the views of a group of young women living in the UK who have a negative view of the calories on menus policy, to understand (a) their perspectives and the objections they raise to policy and (b) the ways they describe the subjective impact of the policy on themselves.

## Methods

### Study design

A qualitative design was implemented using semi-structured interviews with young women fitting the inclusion criteria. Interviews were analysed using reflexive thematic analysis through an inductively oriented framework and a critical realist ontological perspective. The epistemological positioning is contextualist in the sense that the analysis is shaped by the values and practices of the researcher (Braun and Clarke, 2006). Ethics approval was obtained from the Ethics approval was obtained from the University of Bath Psychology Research Ethics Committee, Ref: 23-073.

### Participants and recruitment

The participants were eight White British women between the ages of 18 and 25 years old. The inclusion criteria for the study were specifically women between the ages of 18 and 25 years old who live in the UK, are English speaking and were aware of the addition of calories on menus in the UK. The age range 18–25 was chosen to reflect the age of highest risk for eating disorder onset. The researchers had not intended to recruit only White British women. There were no specific exclusion criteria and women volunteering to take part were not screened for psychiatric/eating disorder history or weight.

Participants were recruited through social media (Instagram) with a recruitment poster, then messaged or emailed the researcher to express their interest. Recruitment materials did not specify that young women should hold a negative view of the policy, however all the young women who volunteered to discuss the topic held a negative view. Eligible participants reviewed an information sheet containing details regarding the study, explaining they would be interviewed about their views on the calories on menus policy. They were asked to indicate informed consent via an online consent form. Upon completion of the study, participants were given a debrief form which provided them with information and signposted relevant psychological resources. Eight participants were recruited to ensure sufficient qualitative information to meet the minimum requirement to achieve thematic saturation (Braun and Clarke, 2021). The sample were felt to hold sufficient informational value to answer a research question with narrows aims, with a group of participants who were highly specific for the study aim and with strong interview dialogue between participant and interviewer (Malterud et al., 2016).

### Measures

A semi-structured interview schedule was developed using 5 open-ended topic questions which each had a variety of prompts to ensure enough data was acquired from each interview. The interview schedule was developed through discussion between the first author, a Masters psychology student and the second author, a qualified clinical psychologist and academic specialising in eating disorders. Questions were designed to be open and not constrained by specific theory, to elicit the views and perspectives of the participants on the policy and any perceived impacts on themselves. The questions explored how the participants felt they were affected by having calories on menus, how they believed those around them were affected by calories on menus and their overall opinion about the presence of calories on menus. This allowed a thorough and varied production of data that fully explored how participants viewed and understood the initiative.

### Procedure

Once consent was received, interviews were conducted synchronously via Microsoft Teams to ensure a high level of data security and privacy. Interviews were conducted by the first author, from June – August 2023. They were video and audio-recorded using the recording function within Microsoft Teams. Once completed, these recordings were immediately transferred to the University’s password protected X drive, before being deleted from both the original device and Teams. Interviews lasted between 20 and 50 minutes and were transcribed manually by the first author into an intelligent verbatim transcript to ensure the participants’ meaning was preserved but unnecessary fillers and irrelevant words were removed. Two weeks from the date of the interview, transcripts were anonymised, and recordings were deleted (in line with participant consent/ data protection considerations) meaning participants were no longer able to withdraw. Participant quotes are used to support analysis conclusions, but all data was stored securely and separately from personally identifiable data, and any names or places mentioned were edited to ensure anonymity.

### Analysis

Analysis was undertaken by the first author, with discussion and supervision from the second author. Following interview transcription, interviews were assigned pseudonyms to replace participant names to protect their identity and were read through multiple times to acquire a thorough, detailed understanding of the data. Initial thoughts and ideas stemming from this were noted down before analysing the data using a thematic analysis approach with the six-step framework suggested by Braun and Clarke (2006) (Table 1).

**Table 1.** Stages of reflexive thematic analysis as outlined by Braun and Clarke (2006) adapted for this study.

Stage	Description
Becoming familiarised with the data	The data was transcribed manually, and transcripts were initially read through twice to gain an in-depth understanding before continuing to the next stage
Coding	The researcher identified relevant codes to the research question which could then be collated and compiled into meaningful groups. Coding was done on paper and each transcript was coded twice to ensure all relevant pieces of data were coded and accounted for
Generating initial themes	Theme ideas were constructed based on the codes that shared key ideas or concepts that provided insight into the research question. This was done on Microsoft Excel where similar codes were combined to help define themes
Developing and reviewing themes	The themes were reviewed and refined, with the researcher deciding whether any changes should be made. Three final themes were generated
Refining, defining and naming themes	The relationship between themes was considered and their place within the overall aim of the paper was defined. Themes were named to help capture what they each represented and what data they captured
Writing up	A final report was then produced to present the findings and analysis of the data by highlighting the themes that were identified. Data extracts were used, and the themes were linked to the broader literature

### Reflexivity and credibility

Both the authors are White British women undertaking this research within the context of an English university. The first author is a young women in the same age group as those interviewed, currently undertaking a Masters qualification in psychology. The second author is in her mid 30s, is a clinical psychologist and academic specialising in eating disorder research and treatment and working as a researcher and clinical tutor in a psychology department. Neither of the researchers were known to the participants before they took part in the research.

To ensure high standards of credibility and reflexivity were met throughout the process, the researcher attended regular meetings with their supervisor and kept notes in a reflective diary. Within the context of the current study, the first author needed to take into consideration their insider status as they are a young woman in the same group as those interviewed. This most likely would have allowed participants to feel more able to talk about their experiences although may have led to bias when interpreting the data. The researcher aimed to combat this bias by considering all points of view and ensuring that during the interviews, they remained impartial and objective during conversation. The second researcher reflected on her position as an eating disorder clinician, with pre-existing negative views related to the potential harms of the policy.

It was understood there was a risk of potential psychological harm to participants due to feelings of anxiety or discomfort around the topic so, time was taken at the end of each interview to ensure the psychological safety and comfort of each participant.

## Results

**Figure 1.**
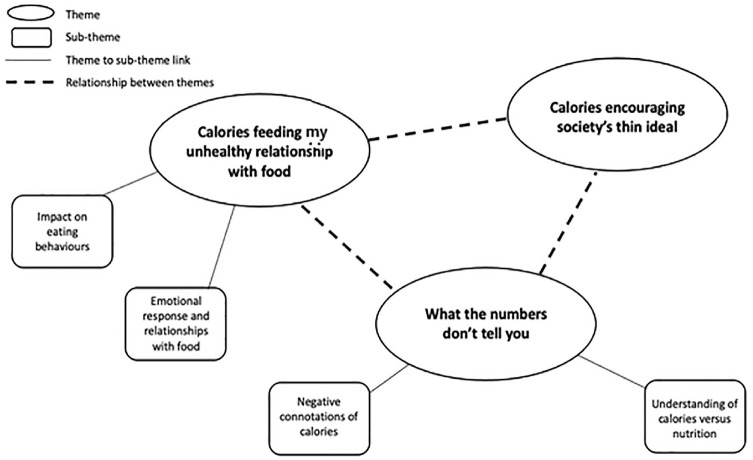
Thematic map.

The data was analysed to explore young women’s perceptions and opinions surrounding the addition of calories on menus in the UK. The negative views of them women on the policy are reflected within the three key themes that were generated. These themes are (1) calories feeding my unhealthy relationship with food, (2) what the numbers don’t tell you, (3) calories encouraging society’s thin ideal. The themes and their subthemes are shown in the thematic map (Figure 1) and are discussed below. All given names are pseudonyms.

### Theme 1: Calories feed my unhealthy relationship with food

The young women described in detail the negative effects of seeing calorie information on menus on their own emotions and behaviour. There were two sub-themes generated within this theme; the first was of the impact they reported on their eating behaviours and the second, was of emotional responses and how their pre-existing relationship with food played a role.

#### Impact on eating behaviours

All participants reported that the addition of calories on menus affected their eating behaviours and thought processes when ordering food. Participants specifically mentioned the changes in their choices when they ate out. Olivia said:

‘*It definitely affected what I ordered… I would even get the kids menu because I would know that there’s less [calories] in there’*.

This highlights the idea that these women described registering the number of calories on a menu but then responded to it with negative behaviours such as ordering less calories than required by a young adult. When changing their orders, participants mentioned how they were doing so because of a negative response they had when seeing the number of calories. Alice said:

‘*I can pinpoint a few times when I’ve changed my order but also it means it makes going out more difficult because I’m anxious… going out is one thing, but going out and having to overthink what you’re going to order as well is another thing’*.

This links to the other subtheme within this theme and the idea that viewing calories on menus results in an emotional response of guilt or anxiety, along with increased aversive rumination on food choice, which in turn affects the food ordered. It was suggested that even if an individual was to make a change to their order as a result of seeing the calories, it wouldn’t be for the ‘right reasons’ (i.e. health) and the issue would therefore not be targeted in a positive, healthy way. Abigail said:

‘… if I’m on my own, it will make me change and rethink what I want or what I thought of ordering, but not for the right reasons’.

This suggests that a view that their changes in ordering behaviour would actually be related and feeding in to unhealthy ideas towards food and calories. This idea was also described by several of the women who felt they had a somewhat positive emotional reaction to calorie information. ‘…*to be honest, [having calories on menus] probably made me quite happy*…*because it meant I could control what I was eating in an unhealthy way even more*’.

And Eleanor said:

‘I think it was kind of like a satisfaction or a “go me” if I had picked something that had a lower number of calories…’

This positive reaction was described in negative terms as indicative of a disordered or unhealthy relationship with food and eating.

#### Emotional response and relationships with food

Participants were also able to identify how they emotionally responded to the addition of calories on menus and it triggered or furthered exacerbated underlying unhealthy emotions and associations within their relationship with food. Charlotte said:

‘Anyone who has a relationship with food, which I think everyone does, they will have a reaction to seeing those numbers on the menu and it does impact how you feel about yourself… it does come linked with guilt’.

Here, Charlotte’s comments outwardly relate to her views on how ‘everyone’ feels, but appear grounded in her own emotional experience. Guilt was a common emotion mentioned by almost all the other participants, highlighting the strong emotional response they would have when confronted with the calories. Emma said:

‘I feel it goes back to guilt and feeling a degree of guilt if I choose an option I don’t deem to be as healthy’.

Most participants repeatedly emphasised the significance of guilt when faced with calories and how individuals believed it played an important role in their emotional response. Guilt here was often linked to making choices which are not ‘healthy’, suggesting some moral significance attached to food decisions.

This emotional experience of guilt was described as acting counter to the intended purpose of the provision of calorie information (to promote healthier food choices). Amelia stated:

‘I can only image it has made more people feel guilty… obviously guilt is not something you want someone to feel but even if the idea was to “guilt-trip” people into being more aware of what they’re eating, I still don’t think that would actually empower [people] to make better choices because if you’re feeling bad about yourself, you don’t think “oh brilliant, let’s go and lead a healthy lifestyle”’.

Anxiety was an emotional response that multiple participants mentioned. Emma said:

‘If I’m expecting it then I’m anxious about it and then I’m anxious about going out and it kind of just snowballs really’.

Going a step further than anxiety, one participant described ‘terror’ related to choosing a meal labelled with a certain number of calories. Olivia said:

‘… it was the first time I had picked a meal which was over 650 calories or something like that and it was terrifying… immediately my mind thought “I’m going to put on weight”’.

Participants also brought up the idea that having calories on menus negatively impacted their experience of eating out as a whole, something that they previously would enjoy or have had positive associations with. Freya said:

‘… it has completely flipped going out for food from something fun and enjoyable to like just looking at a number and being like “yeah that’s what I’ll have”. Rather than “what do I actually want”’.

Here Freya describes an experience of the inclusion of a calorie number creating a disconnect with her sense of what food she desires and enjoys. The presence of calorie information, even after ordering has finished, was described as having a negative impact across the whole meal. Abigail described:

‘I’m starting to realise that when I’m eating out, I should enjoy my food as it is a treat, but it ends up ruining the whole experience. It’s always at the back of my mind knowing about the calories there and it means I don’t appreciate the meal’.

Several participants also commented on the competitive nature of eating decisions that they felt, related to their poor body image and how this was exacerbated by knowing the caloric value of their meals when eating out with peers. Emma said:

‘… when you go out for a meal with friends, *one* of the times I might feel more insecure about my body is if I think “oh everyone else has ordered meals which are 500 calories but mind is 800 calories” and then it kind of feeds into that “oh I’m bigger than everyone, I’m fatter than everyone else” which goes into that idea of body image and my self-confidence which again, is a very unhealthy thought process’.

Similarly, Alice mentioned her comparison to those around her:

‘… it would also be the comparison of what everyone else is eating… “you look like you’re really overindulging compared to the people around you”’.

In these accounts, participants appeared to blame the inclusion of concrete numerical calorie information for increasing their ability to compare their intake to others and also the presence of others for amplifying the impact of calorie information on their emotional state. This increased comparison was described as activating self-critical thoughts related ‘fatness’ or ‘greed’.

### Theme 2: What the numbers don’t tell you

This theme highlighted the views of this group of women on the meanings and associations with calories that they felt are ingrained within society. There were two sub-themes generated; the negative connotations linked with the concept of ‘calories’, and the comparative knowledge of calories versus nutrition.

#### Negative connotations of calories

Participants emphasised an unhealthy, negative connotation of ‘calories’. They described their view that there was a widespread belief of calories being a negative thing, rather than this belief being something specific to them. Eleanor said:

‘… I think from my experience… the notation of calories is more of a negative thing for most people’.

Emma also said:

‘… the way society talks about calories, an average person’s perception of calories is going to bring up more negative things than just viewing it as “oh this is a specific energy input”’.

The participants described ‘calories’ as a concept with broader cultural and emotional significance than just their scientific meaning. Whilst participants suggested that society as a whole views calories quite negatively, individuals also highlighted that they believed young women had stronger negative associations with calories than other groups of people. Freya said:

‘… my brother and dad, I don’t think they’re really bothered by [the number of calories] … whereas with my female friends and my sister, and just women in general, I would say they have a much more negative view of it’.

Individuals also spoke about why they believed there was this negative association surrounding calories. Amelia said:

‘It’s this idea that society has encouraged that if something has more calories, that’s bad. If something has less calories, that’s good. It doesn’t matter if you’re trying to lose weight or not, those are just the ideas that we have been raised with’.

This idea that higher calorie options are ‘bad’ whilst lower calorie options were ‘good’ was mentioned by most participants. These ‘bad’ and ‘good’ meanings were described as consistent regardless of someone’s real nutritional needs or the broader properties of the food. The reference to ‘good’ and ‘bad’ echoes back to the discussion related to guilt and considerations of the moral nature of food choices.

#### Understanding of calories versus nutrition

A key sub-theme that arose was the idea that there was often much more emphasis and focus on calories than on wider nutritional information and value. This was viewed as a negative societal tendency by the women interviewed. The inclusion of calorie information on menus was described as exacerbating and amplifying this focus. Participants emphasised how this misunderstanding was due to a lack of education and the significance society places on caloric value. Charlotte said:

‘We’re not well educated [about calories] either… we should be taught about healthy diet and balance and why food is to fuel ourselves and to make ourselves healthy as well as to be enjoyed’.

This was a common theme amongst participants and Amelia also commented on the need for this to be corrected and for society to refocus its attention onto nutrition rather than the number of calories:

‘… I think that there should be more of a focus on the general health of a meal or of food in general. So, I think that calories aren’t the “be all or end all” and I think there is so much emphasis put on the numerical value of food and the number of calories, but we don’t talk about the fibre and all the micronutrients, and if you’re hitting your vitamins and minerals… just looking at calories is too simplistic and it doesn’t take into account all the other factors that could make a food “healthy”’.

Participants also believed that this stronger reliance on calories over nutrition was much more common amongst young women in comparison to other groups. Olivia said:

‘I think there is a certain audience that are completely focused on calories and that is probably the younger girls stereotypically…’

Even though participants were aware of this unequal balance of importance placed on calories over nutrition, they still felt that having that knowledge wasn’t enough to combat the negative associations they had with calories. Alice said:

‘I now know those numbers aren’t accurate… I know it’s not accurate for the same meal… but it still affects me’.

This emphasises the disconnect between participants described knowledge that calories aren’t as significant as they are made out to be, with their ongoing felt sense of their significance. They linked this felt sense to the deeply ingrained nature of attitudes to calorie information.

### Theme 3: Calories encouraging society’s thin ideal

This theme explored how these women felt thinness is encouraged amongst young women, and the pressure participants felt societal thinness ideals put on them. These ideals were described as increasing the negative impact of calorie information policies on young women in particular.

All those interviewed emphasised that they believed that young women would be the most likely to be impacted by the addition of calories on menus and that there were significant differences between how men and women would receive calories on menus. Charlotte said:

‘I also think that a lot of it unfortunately, is going to come down to gender… more girls on average have been exposed to these negative messages about eating than boys… they’ve been given those messages about weight loss and the linkage between being thin and therefore attractive’.

Charlotte here clearly links young women’s increased exposure and internalisation of the thin ideal to the nature of young women’s response to calorie information on menus.

Participants also described society as encouraging participants to strive for thinness and beauty through dietary and calorie restriction. They also emphasised how women are exposed to this message from a very young age. Abigail said:

‘I mean it’s drilled into young women, the association between attractiveness and thinness, like from a very young age, and how do we become thin? We lower our calories because that’s the end goal, to be eating the lowest number of calories possible so that you don’t get fat and therefore become unattractive. Having calories readily available when eating out just encourages this thought process’.

Freya also said:

‘I feel like women are more affected by initiatives such as this because our society has it deeply ingrained to make women feel more self-conscious about their appearance and their diet’.

Here the provision of calorie information on menus is described as activating these beliefs and ideals about thinness, restriction, appearance and diet, with the implication that this drives both unhealthily restricted food choices and also negative emotional reactions to calorie information.

Some of the women drew links to the increased rates of disordered eating and eating disorders in girls and women, and implicated societal body representations to both increased disordered eating and the perceived increased negative impact of calories on menus on young women. Eleanor said:

‘The media representation of female bodies and the fact that they’re more socially active but then also more likely to have had a disordered relationship with food… I think calories on menus would disproportionately affect girls the same way that eating disorders themselves disproportionately affect girls’.

Here, it is clear that it is not only those with eating disorders, but all young women, who are to be considered by this group to be at risk of harm from calorie information, as a result of the sociocultural context they find themselves in.

## Discussion

This is the first qualitative study to focus on the views and described experiences of young women in response to the calories on menus policy in the UK. The young White British women interviewed described negative views of the policy which appeared to be grounded heavily in their own negative emotional responses to viewing calorie information on restaurant menus. These young women situated their responses, which they tended to describe as the responses of ‘all young women’, or ‘everyone’, within their description of societal meanings and ideals related to ‘calories’, ‘thinness’, ‘attractiveness’, ‘fatness’, body image, dieting and ‘heathy eating’. The themes generated from the interviews were: (1) calories feeding my unhealthy relationship with food, (2) what the numbers don’t tell you and (3) calories encouraging society’s thin ideal.

The first theme identified the young women’s descriptions of their own experiences of strong psychological effects as a result of calories being added to menus. Participants described a significant change in their eating behaviours when they ate out because of the calories on menus. They attributed this change, which they viewed as unhealthy and indicative of an unhealthy relationship with food, to increased negative rumination on food choices and sometimes intense emotional responses (guilt, anxiety, ‘terror’, loss of enjoyment) to viewing the calorie information on menus.

Whilst the aim of this policy is to inform and educate so individuals would want to make healthier decisions (Jeacle and Carter, 2023), participants emphasised that any changes in food choice were down to feelings of guilt and feeling pressured rather than actively wanting to look after their health. The experience of guilt, and references to ‘goodness’ and ‘badness’ suggest a moral dimension to decision making around food choice for this group. Qualitative research with Irish adolescents has highlighted the role of guilt, and also social desirability and peer presence, in guiding internal conflict around food choice (Daly et al., 2024). The women in this study, aged under 25, can be considered somewhat as older adolescents. The impact of peer presence and comparison in amplifying the negative emotional impact of viewing calories was also raised by the women in this group. The young women described positive emotions were linked with choosing lower calorie options whilst negative emotions were experienced because of higher calorie options being chosen. These impacts defined the eating out event for participants. Initially they spoke about enjoying going to eat out, but feeling such strong emotional effects led them to choose options they did not actually want and induced feelings of anxiety.

These ideas link to the second theme, in which the young women described society as overvaluing calorie information while ignoring the wider health value of food. All participants spoke about how they believed there was a lot of miseducation surrounding calories and a general negative association with them. They described what they felt was a pervasive view amongst all people, and particularly young women, that less calories equals good, and more calories equals bad. Participants also believed these beliefs were stronger amongst women in comparison to men which is supported by studies identifying gender differences in attitudes to food (Fagerli and Wandel, 1999; Striegel-Moore et al., 2009). They linked this to societal messaging about dieting and thinness which they felt women were significantly exposed to.

The young women described a tension of understanding the difference between calories and general nutrition, but still focussing heavily on the importance of calories. Studies have identified the importance of other nutritional factors in foods when trying to lose weight (Finaret and Masters, 2019). Participants themselves emphasised this, yet still mentioned their inability to shift their attention from the number of calories. The women suggested this overfocus reflected a deeper issue regarding society’s view of calories and the level of significance they are held to.

The third theme links to this and the over-emphasised importance placed on thinness in women. Participants felt that society had encouraged thinness amongst women since a very young age, which is well supported by studies (McCarthy, 1990; Wiseman et al., 1992) and linked this to how they felt young women received calories being on menus. Participants felt that having calories readily available on menus was continuing to encourage the idea that being thin was equivalent to beauty in women and was therefore furthering these dangerous stereotypes. Participants linked the thin ideal to the behavioural changes and strong emotional responses they described ‘people’ experiencing when viewing the calories, due to it being a message they had been exposed to from a young age. They believed that the initiative disregarded their possible experience with this. For this group of women, conscious awareness of the sociocultural basis of these ideals (and knowledge of the incompleteness of calorie information) was not enough to inoculate them against their felt emotional impact.

### Limitations

The results of this research must be contextualised within the narrow nature of the participant group. Despite the intention to recruit young women of any ethnicity, race and socio-economic background, all participants identified as White British. This has significant implications for the transferability of findings, as eating and beauty norms differ between different groups. Women from different cultural and ethnic backgrounds may therefore respond differently to the calories on menus policy. The researchers did not enquire about factors such as past or present psychiatric history (including eating disorder), education level or economic background of participants. The group had all volunteered to discuss the calories on menus policy with a researcher, and several acknowledged the fact that they had chosen to do so as a result of their sometimes strongly held opposition to the policy. The experiences and views of this group therefore cannot be considered fully transferable to ‘all young women in the UK’.

The study has a small sample size, eight women. Literature regarding qualitative research methods has suggested different minimum sample sizes, with rationales related to various kinds of ‘saturation’ and ‘information value’ of the sample, or even questioned the value of the concept of data saturation within reflexive thematic analysis (Braun and Clarke, 2021; Malterud et al., 2016; Wutich et al., 2024). It is our view that the analysis in this paper contributes useful meaning to the discussion related to the calories on menus policy. However, it is clear that research with a larger and more diverse number of participant interviews could identify more themes and meanings, with increased potential for transferability of findings.

The insider positioning of the researcher as a young White British women, living in the UK, will have had potential to influence the analysis, as would the second authors position as an eating disorder clinician. Use of a reflective journal, regular supervision, a deliberate stance of openness to viewpoints, and the careful grounding of themes in the data, were used to consider and minimise the impact of the researchers own experiences and views as a source of potential bias, however it would not be possible for the researcher to completely ‘bracket’ their self from the analysis. Braun and Clarke (2019) emphasise the active, reflexive, subjective, analytic role of the researcher in reflexive thematic analysis. Review of the reflective journal regularly through analysis and write up, and prior to research supervision, allowed the first author to notice and interrogate occasions where their own implicit and explicit meanings and assumptions were apparent in their analysis, and to return again to review data and consider their interpretation.

### Implications and future research

This study has a number of key implications for researchers and health policy makers. The negative views and objections of these women to the calories on menus policy appeared highly informed by, and potentially indicative of, their own experiences of anxiety, worry, guilt and loss of enjoyment related to seeing calorie information on menus, which for some women were linked to unhealthy dietary restriction (such as ordering from the children’s menu). Given that negative opinions on the policy are very common amongst young British women (YouGov, 2022), as is poor body image, it is plausible that this suggests that emotional distress in response to seeing calorie menus is also common in this group. When developing a national public health policy, cost/benefit analysis is required and groups other than the intended target will still be receiving the information being given. Policy makers should carefully consider the risks of accidental harm posed by this policy. With increasing numbers of eating disorders amongst young women especially, the potential for a link between viewing calorie information on menus, emotional distress, and unhealthy dietary restriction, should be considered in further research. While not all women will have this experience, the potential for causing frequent emotional distress to some young women is also a potential harm in its own right, albeit one with less cost and significance. The relevance of less significant harms such as this must be weighed up and considered alongside growing evidence about any benefit of this policy. Initial research suggests that the introduction of calorie information on menus in UK restaurants has not resulted in significant changes to food choice and ordering patterns for the general public (Polden et al., 2024).

Future research could investigate the emotional and behavioural responses of wider groups of young women to viewing calorie information on menus, including using experimental and population level designs. Studies could also consider impacts in other groups who may also be considered at higher risk related to sociocultural image and diet pressures and disordered eating.

### Conclusions

Overall, the analysis demonstrated some of the personal emotional responses and reasoning related to the negative views of a group of young White British women about the calories on menus policy. The young women’s negative opinion on the policy appeared heavily grounded in, and indicative of, their own sometimes intense negative emotional responses (particularly guilt and anxiety) to viewing calorie information. The women linked these responses to sociocultural meanings and ideals related to thinness, fatness, calories, food choice and dieting, which they felt young women were particularly exposed to. The implications of these results should be considered in the context that negative views about this policy are common amongst young women in the UK, and the developing evidence base regarding whether this policy has had any of the intended positive impact on health and obesity rates in the UK population. It is important to understand how groups are affected by public health initiatives and, to the author’s knowledge, there is little on how calories on menus impacted young women. With this being an especially vulnerable group when it comes to eating behaviours and thought processes related to this, the importance in understanding these perspectives is highlighted.
